# Benefits of secretion clearance with high frequency percussive ventilation in tracheostomized critically ill patients: a pilot study

**DOI:** 10.1007/s10877-022-00970-7

**Published:** 2023-01-06

**Authors:** Eugenio Garofalo, Serena Rovida, Gianmaria Cammarota, Eugenio Biamonte, Letizia Troisi, Leonardo Cosenza, Corrado Pelaia, Paolo Navalesi, Federico Longhini, Andrea Bruni

**Affiliations:** 1 Anaesthesia and Intensive Care, Department of Medical and Surgical Sciences, Magna Graecia University, Catanzaro, Italy; 2 Department of Intensive Care Medicine, St George’s University Hospital, London, UK; 3 Department of Anaesthesia and Intensive Care Medicine, University of Perugia, Perugia, Italy; 4 Pulmonary Medicine Unit, Department of Health Sciences, Magna Graecia University, Catanzaro, Italy; 5 Anaesthesia and Intensive Care, Department of Medicine—DIMED, Padua Hospital, University of Padua, Padua, Italy; 6 Intensive Care Unit, Department of Medical and Surgical Sciences, Mater Domini University Hospital, Magna Graecia University, Viale Europa, 88100 Catanzaro, Italy

**Keywords:** Cough, Acute respiratory failure, High-frequency percussive ventilation, Chest physiotherapy, Lung aeration, Electrical impedance tomography

## Abstract

Clearance of secretions remains a challenge in ventilated patients. Despite high-frequency percussive ventilation (HFPV) showing benefits in patients with cystic fibrosis and neuromuscular disorders, very little is known about its effects on other patient categories. Therefore, we designed a physiological pilot study investigating the effects on lung aeration and gas exchange of short HFPV cycles in tracheostomized patients undergoing mechanical ventilation. Electrical impedance tomography (EIT) was recorded at baseline (T0) by a belt wrapped around the patient's chest, followed by the HFPV cycle lasting 10 min. EIT data was collected again after the HFPV cycle (T1) as well as after 1 h (T2) and 3 h (T3) from T0. Variation from baseline of end-expiratory lung impedance (∆EELI), tidal variation (TIV) and global inhomogeneity index (GI) were computed. Arterial blood was also taken for gas analysis. HFPV cycle significantly improved the ∆EELI at T1, T2 and T3 when compared to baseline (p < 0.05 for all comparisons). The ratio between arterial partial pressure and inspired fraction of oxygen (PaO_2_/FiO_2_) also increased after the treatment (p < 0.001 for all comparison) whereas TIV (p = 0.132) and GI (p = 0.114) remained unchanged. Short cycles of HFPV superimposed to mechanical ventilation promoted alveolar recruitment, as suggested by improved ∆EELI, and improved oxygenation in tracheostomized patients with high load of secretion.

*Trial Registration* Prospectively registered on www.clinicaltrials.gov (NCT05200507; dated 6th January 2022).

**Supplementary Information:**

The online version contains supplementary material available at 10.1007/s10877-022-00970-7.

## Introduction

Physiological processes involved in the clearance of the airways are compromised during mechanical ventilation (MV). On one hand, the endotracheal tube (ETT) itself alters the mucociliary function [1]; on the other hand, sedation inhibits the physiological cough reflex. Nonetheless, the cold and dry air-oxygen delivered during MV alters the bronchial epithelium function preventing the mucus transit to upper airways [2–4]. As a result, therisk of ventilator associated pneumonia (VAP) and lung atelectasis is increased [2, 5, 6].

While that ETT suction alone results in a partial airways clearance [7], the combination of suctioning and chest physiotherapy leads to several physiological and clinical benefits [8] including re-expansion of atelectatic portions of the lung, improvement of the respiratory system compliance [9] and expiratory flow rates [10] as well as reduction of VAP [11].

Over the years, novel approaches have been proposed to ease the clearance of the airways. In this regard, high-frequency percussive ventilation (HFPV) demonstrated its ability to mobilise secretions in patients affected by cystic fibrosis [12, 13] and neuromuscular disease [14]. In line with these previous results, we designed a physiological study to investigate the benefits of HPFV when superimposed to conventional MV in tracheostomized patients with a high amount of secretions. Changes in lung aeration following the application of HFPV was the primary aim of the study. Secondary aims were the changes in distribution of ventilation within different regions of the parenchyma and the gas exchange variation.

## Materials and methods

This observational study was conducted in the Intensive Care Unit of the “Mater Domini”—University Hospital of Catanzaro (Italy) after local Ethical committee approval (Ethical Committee Approval number 314 dated 16th September 2021). Written consent for publication of individual details including images was obtained from the patients or the next of kin.

This trial was compliant with the consolidated standards of reporting trials (CONSORT) reporting guidelines and was prospectively registered (NCT05200507; www.clinicaltrials.gov, dated 6th January 2022).

### Population

Study group comprised of adult patients (≥ 18 years/old) meeting the following criteria: (1) MV through tracheostomy for more than 48 h with heated humidification and (2) Requiring two or more suctions/hour within 8 h. These patients were classified as hypersecretive patient [7]. Criteria for study exclusion were the following: (1) Life-threatening cardiac arrhythmias or signs of ischemia; (2) Pneumothorax; (3) Hemoptysis; (4) Acute spinal injury; (5) Need for vasopressors; (6) Traumatic brain injury; (7) Pulmonary embolism; (8) Recent chest trauma or burn; (9) Recent surgery; (10) Pregnancy; (11) Enrolment in other research protocols and (12) Denied consent.

As per our standard practice, suction occurred only if auscultation revealed secretions in the upper airway or the airway pressure waveform indicated fluid in the system and/or if the peak airway pressure increased [7, 15].

### Study protocol and data acquisition

Similarly to our previous protocols [7, 16], a silicon EIT belt with 16 electrodes was placed at the level of the 4th and 6th rib space. The belt was connected to an EIT device (PulmoVista 500; Draeger Medical GmbH, Lübeck, Germany) and its position was marked on the skin to avoid displacement [7, 16, 17]. Noteworthy, the EIT belt was never moved or removed from the patient and its position checked before every single recording, to assure the recording quality [7, 16, 17]. In addition, pulsating air suspension mattress was switched off during the study protocol to minimize possible electrical interferences, baseline drifting, step-like and/or spike-like signal [18, 19].

A heated humidifier (MR850 heated humidifier; Fisher & Paykel, Auckland, New Zealand) was connected to the breathing circuit and set at a fixed temperature of 37 °C with relative humidity at 100% (*i.e.,* 44 mg/L of absolute humidity). Ventilation data (V500; Draeger Medical GmbH, Lübeck, Germany) were transmitted to the EIT device by a RS232 interface.

Analgesia and sedation regime as well as ventilation mode (Pressure Support) remained unmodified within the whole trial. Ventilator variables including inspiratory pressure support (PS) and positive end expiratory pressure (PEEP) were different in each patient but once defined remained unchanged throughout the whole study duration. No further fluid challenge (isotonic or hypertonic saline) was administered throughout the study protocol. After initial suction, the EIT at baseline (T0) was recorded. Following EIT registration, HFPV cycle (Travel Air, Percussionaire, Bird Technologies, Sandpoint, ID, USA) was superimposed for 10 min at the maximum oscillation frequency (500 pulses per minute) and highest pulse amplitude. Straight after the cycle, suction was performed again, EIT data were then recorded for 10 min (T1). In the same manner, measurements were registered after 1 h (T2) and after 3 h (T3) from baseline (T0) [7]. ETT suctioning was delivered by a closed aspiration system (KimVent, Turbo-Cleaning Closed Suction System, Kimberly-Clark, Roswell, GA, USA).

The protocol was interrupted if one or more of the following conditions were encountered: (1) Hemodynamic instability; (2) Life-threatening arrhythmias or electrocardiographic ischemic changes; (3) Oxygen saturation (SatO_2_) drop below 88%, as assessed by pulsoxymetry [7].

### Data analysis

Comprehensive information about EIT data acquisition and analysis is provided in the Supplemental Material (Supplemental Material). Briefly, we computed the tidal impedance variation (TIV) as the difference of impedance between the end of inspiration and expiration [20], changes in end-expiratory lung impedance (∆EELI, mL) at T1, T2 and T3 from baseline (T0), which is a surrogate measure of the end-expiratory lung volume [17, 20, 21] and the inhomogeneity index (GI) to assess the gas distribution within the lung [17, 22]. We also defined two contiguous regions of interest of the same size (ventral and dorsal) and computed TIV, ∆EELI for both [17, 21].

We also calculated the variations of PaO_2_/FiO_2_ (∆PaO_2_/FiO_2_) consisting of the difference between PaO_2_/FiO_2_ value at T0 and the PaO_2_/FiO_2_ registered at each protocol step from T1 to T3.

### Statistical analysis

We assumed all our data as non-parametric. Continuous variables were compared with the Friedman tests for repeated measures analysis of variance by ranks. Post-hoc Bonferroni test was applied for pairwise multiple comparisons, when indicated. Statistical analysis was performed using the Sigmaplot v. 12.0 (Systat Software Inc., San Jose, California).The correlation between ∆PaO_2_/FiO_2_ and ∆EELI at T1, T2 and T3 was assessed through the coefficient of correlation for repeated measures and results reported as R_rm_ [95% Confidence Interval] on a web and standalone application [23]. We considered significant two-sided p values < 0.05.

## Results

Fifteen adult patients were enrolled consecutively from January 2022 to April 2022 and all of them completed the study protocol. Demographic, anthropometric and clinical features of the patients are reported in Table 1. The mean (Standard Deviation) PEEP was 8 (2) cmH_2_O and the mean inspiratory PS was 10 (2) cmH_2_O. Mean value of inspired Oxygen fraction (FiO_2_) was 0.39 (0.03).

**Table 1:** Characteristics of the population at protocol inclusion

Patient	Gender	Weight (kg)	Height (cm)	Age (years)	SAPS-2	SOFA	Reason of ICU admission
1	M	78	174	55	22	4	Acute respiratory distress syndrome
2	F	60	158	54	24	7	Septic shock
3	M	75	180	67	37	6	Cardiac arrest
4	F	68	165	48	32	6	Intraparenchymal cerebral haemorrhage
5	F	70	165	74	37	7	Acute respiratory distress syndrome
6	M	87	182	62	32	4	Cerebral ischemia
7	M	68	178	32	20	4	Acute respiratory distress syndrome
8	M	90	172	78	44	7	Septic shock
9	F	58	160	72	48	7	Cardiogenic shock
10	M	50	170	31	20	3	Acute respiratory failure in multiple Sclerosis
11	M	75	170	59	29	5	Acute on chronic respiratory failure
12	F	73	166	48	47	7	Coma in thrombocytopenic thrombotic purpura
13	F	78	164	67	29	4	Acute respiratory distress syndrome
14	F	82	168	64	32	4	Acute respiratory distress syndrome
15	M	58	172	23	22	4	Acute on chronic respiratory failure in duchenne dystrophy
Mean (SD)		71 (11)	170 (7)	56 (17)	32 (9)	5 (1)	

*SAPS-2* simplified acute physiology score 2, *SOFA* sequential organ failure assessment score, *ICU* intensive care unit

**Fig. 1:**
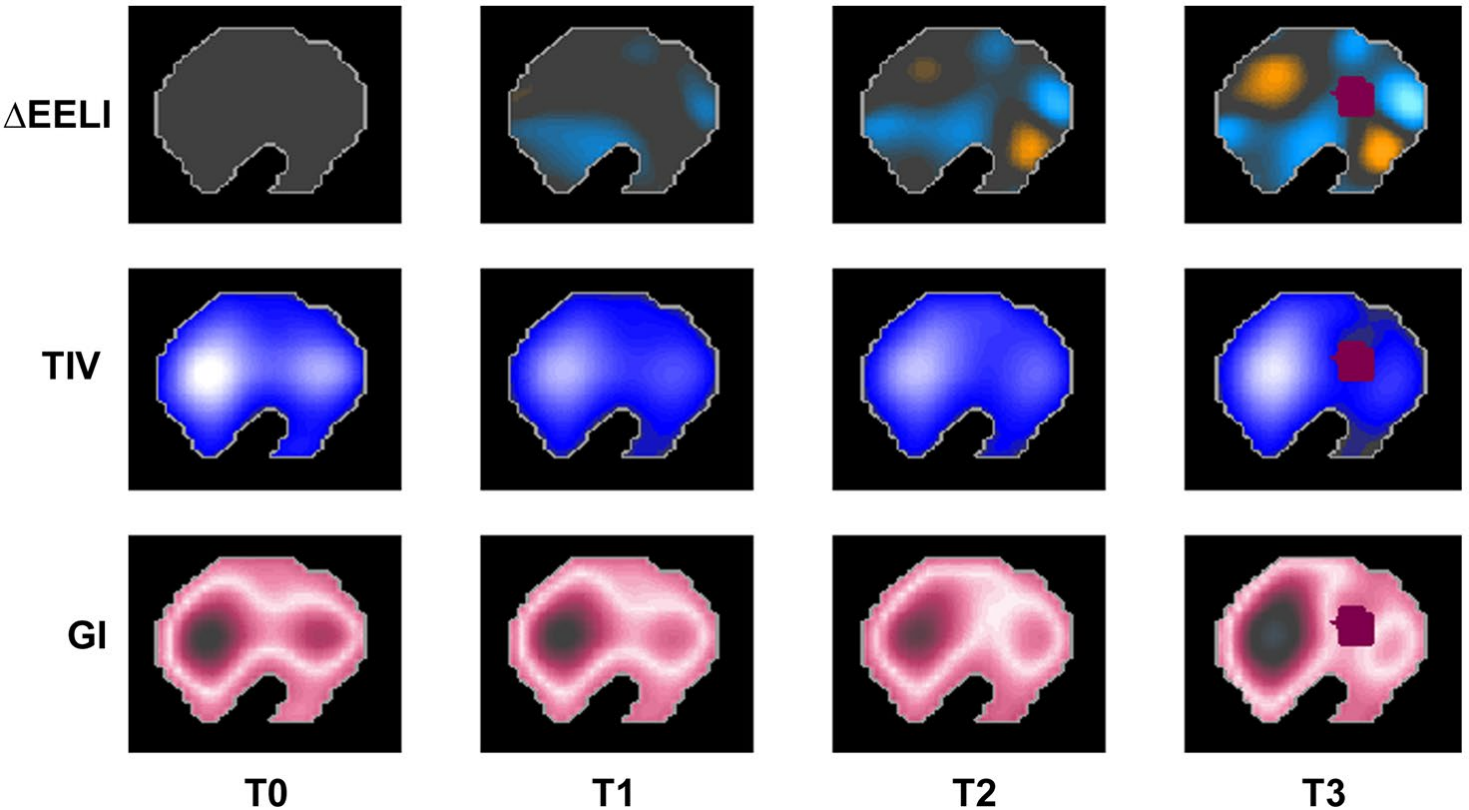
EIT images from one representative patient. Examples of EIT images collected from one representative patient; ∆EELI, TIV and GI are shown at baseline (T0), T1, T2 and T3. Yellow and blue codes indicate EELI loss and increase, respectively. A blue scale code indicates tidal variation, whereas pink scale code indicates lung inhomogeneity. The darker is blue or pink, the highest are the tidal variation or inhomogeneity, respectively. Of note, at T3 purple code represents a not-ventilated area (i.e. the heart). In this patient, EELI significantly increased at T1 (60 mL), T2 (148 mL) and T3 (193 mL). TIV and GI did not significantly change throughout the study protocol. ∆EELI changes from baseline (T0) of end-expiratory lung impedance, TIV tidal impedance variation, GI global inhomogeneity index

Figure 1 depicts EIT images from one representative patient (see figure legend for explanation). As shown in Table 2, the TIV did not change after the application of HFPV neither globally (p = 0.132), nor in dorsal (p = 0.114) and ventral (p = 0.125) lung regions. Compared to baseline (T0), ∆EELI improved significantly straight after HFPV cycle (T1). Similar results were also observed after one (T2) and 3 h (T3) from the baseline (T0) prior to HFPV cycle. To note, alveolar recruitment was achieved on both dorsal and ventral lung regions. The GI was not modified after the cycle (p = 0.114).

**Table 2:** Electrical impedance tomography

Data	T0	T1	T2	T3	P value
TIV (ml)	400 [333; 423]	366 [297; 602]	420 [325; 620]	364 [274; 468]	0.132
Dorsal	164 [106; 215]	153 [109; 316]	166 [116; 280]	143 [109; 254]	0.114
Ventral	206 [169; 266]	213 [164; 328]	239 [181; 333]	214 [156; 240]	0.125
∆EELI (ml)	0 [0; 0]	258 [56; 332]^a^	276 [148; 451]^b^	290 [184; 412]^c^	< 0.001
Dorsal	0 [0; 0]	154 [72; 202]^a^	143 [42; 327]^b^	133 [107; 232]^c^	< 0.001
Ventral	0 [0; 0]	73 [− 16; 157]^a^	76 [38; 173]^b^	77 [58; 206]^c^	< 0.001
Inhomogeneity index	70 [41; 92]	55 [40; 69]	60 [38; 72]	67 [39; 73]	0.114

All data are expressed as median [25th–75th percentile]

*T0* baseline assessment, *T1* assessment soon after the end of the treatment, *T2* assessment 1 h after the end of the treatment, *T3* assessment 3 h after the end of the treatment, *TIV* tidal impedance variation, *∆EELI* difference of end-expiratory lung impedance from T0

^a^p < 0.05 T1 vs. T0

^b^p < 0.05 T2 vs. T0

^c^p < 0.05 T3 vs. T0

**Fig. 2:**
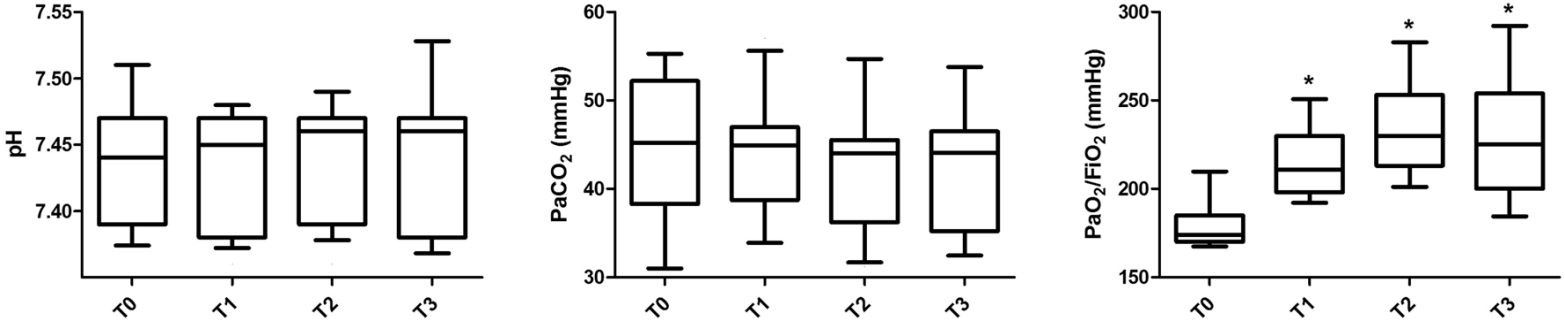
Arterial blood gases. Data (left to the right) in the box (plots) show the pH, the arterial partial pressure of carbon dioxide (PaCO_2_) and the ratio between arterial partial pressure to inspired fraction of oxygen (PaO_2_/FiO_2_) at each time frame of the study protocol. The bottom and top of the box indicate the 25th and 75th percentiles, respectively. The horizontal band close to the middle of the box represents the median, whereas the ends of the whiskers represent the 10th and 90th percentiles. Statistically significant p values are also reported. *p < 0.001 in comparison with T0

Figure 2 reports the arterial blood gases obtained at each step of the protocol, from T0 to T3. Detailed data are shown in Table E1 in the ESM. The pH (p = 0.226) and the PaCO_2_ (p = 0.114) did not change after the HFPV cycle, while PaO_2_/FiO_2_ significantly improved along the protocol. Specifically, PaO_2_/FiO_2_ was 174 (171; 185)) at T0, 211 (198; 225), p < 0.001) at T1, 230 (213; 251), p < 0.001) at T2 and 225 (203; 251), p < 0.001) at T3.

**Fig. 3:**
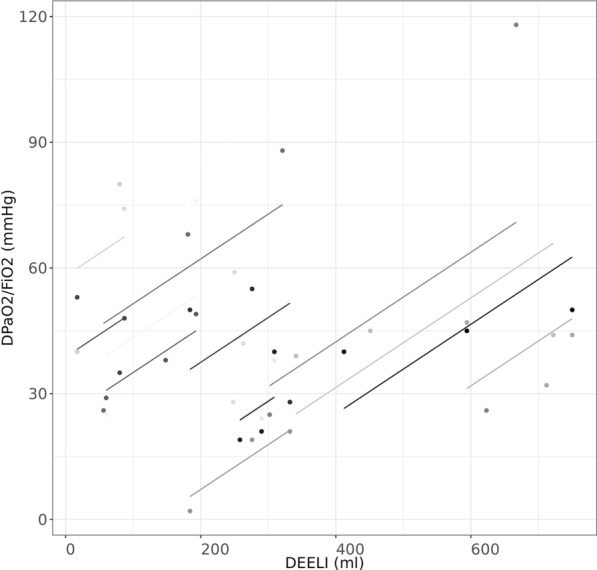
Association for repeated measures between ∆EELI and ∆PaO_2_/FiO_2_. The relationship between the changes in end-expiratory lung impedance (∆EELI) and difference of PaO_2_/FiO_2_ from baseline (∆PaO_2_/FiO_2_) are depicted (R_rm_ = 0.53, 95%CI (0.222, 0.747); p = 0.002). Every single dot represents one measurement for every patient, whereas every single line represents the association between variables within each patient

Compared to baseline (T0), ∆PaO_2_/FiO_2_ was observed at each protocol step. Specifically, from T0 to T1 ∆PaO_2_/FiO_2_ was 28 [25; 45] mmHg, from T0 to T2 it was 45 [40; 68] mmHg and from T0 to T3 it was 44 [26; 76] mmHg. As shown in Fig. 3, the ∆PaO_2_/FiO_2_ correlated with the increase in ∆EELI (R_rm_ = 0.53, 95%CI (0.222, 0.747); p = 0.002).

No modifications of vital parameters (heart rate, mean arterial pressure and respiratory rate) were observed (see Table E1 in the ESM).

## Discussion

Our study demonstrated the application of the HFPV cycle during MV in hypersecretive tracheostomized patients promotes alveolar recruitment and improves oxygenation. Both benefits are remarkable and last up to 3 h after the treatment. Besides these physiological advantages, HFPV cycles also have a positive impact on reducing the manual work load of nursing staff and physiotherapists [7].

The management of airways secretions gained progressive attention in critically ill ventilated patients as it may lead to complications such as VAP and extubation delay [24, 25]. Despite several methods being suggested, the clearance of deep airways remains a challenge. Mechanically assisted cough devices mimicked the cough process through positive and negative air pressure which showed promising outcomes in the paediatric population and those patients with neuromuscular disorders. However, these devices can only reach upper airways [26]. Another alternative technique involved oscillating devices: inspiratory and expiratory chest oscillations at high rate promote the migration of mucus towards the bronchial tree where it was cleared by suction [7]. Despite the benefit, evidence in literature remains contradictory as the superior effectiveness of these devices to chest physiotherapy has not been demonstrated yet [27].

Among the group of high Frequency Ventilation, HFPV is mainly applied to patients with inhalation injury or Acute Respiratory Distress Syndrome. It improved oxygenation and enhanced mobilisation of secretions from lower to upper airways [28]. Given this, Clini et al. investigated the effects of HFPV in extubated and tracheostomized patients during weaning for the first time [29]. Specifically, forty-six patients were randomised to receive either conventional chest physiotherapy or the combination of HFPV together with physiotherapy. Results confirmed the superiority of HFPV and physiotherapy for secretion clearance and improvement of gas-exchanges [29]. Similarly, Tsuruta et al. explored the effects of HFPV superimposed to MV in ten obese patients. HFPV was continuously applied from study enrolment till the beginning of the weaning process [30]. Gas exchange and lung dynamic compliance was better after 3 h, assuming a potential recruitment effect of HFPV [30]. In a small sample of patients with moderate-to-severe Acute Respiratory Distress Syndrome, Godet et al. reported that HFPV improves lung aeration and gas exchange, by recruiting the dorsal lung regions while assuring a protective and gentle ventilation[31]. A previous study conducted by our research group assessed the effects of high-frequency chest wall oscillation (HFCWO) in ventilated patients with significant and minimal secretion load. Increase in lung aeration was noted only in patients with high secretion load, however there was no gas exchange improvement. An explanation for these results were addressed to the amelioration of the ventilation-perfusion mismatch rather than reduction of the intrapulmonary shunt [7]. To note, the benefit on lung aeration did not last beyond 3 h from the HFCWO cycle [7].

In addition, we did not record any difference of GI after the one HFPV cycle in our population. The GI is a parameter that assess the inhomogeneity of air distribution within the lung [32]. A higher GI implies more heterogeneous spatial ventilation in the lung (i.e. a “sicker” lung) [33]. Several studies have shown that the application of a personalized PEEP to avoid alveolar derecruitment guarantees lower GI values in patients with acute respiratory failure [34–36]. However, when PEEP is set guided by GI, its best value may be overestimated [37]. Beside the relationship between GI and PEEP, we used GI to assess if the intra-tidal distribution and lung inhomogeneity was different before and after HFPV application. In fact, the recruitment induces by the application of HFPV may have also reduced the intra-tidal distribution and the lung inhomogeneity. However, this event did not occur in our patients, probably because they were not so acute and severe as in previous other studies [38–40].

Divergence between our current results and previous ones could be partially explained by two aspects: the ventilation settings and the study population we had enrolled. Unlike previous trials where MV was delivered throughout ETT [7], our patients underwent MV via tracheostomy. Since patients with tracheostomy are generally those with longer stay in ICU and worse gas exchange, their weaning process is usually more challenging [7, 41]. As a result, the amount of recruitable lung can remarkably vary between tracheostomized and intubated patients.

Given the benefits of the HFPV cycle lasting up to 3 h, we extended its application beyond our research protocol. Specifically, HFPV was delivered for 10 min every 6 h till 48 h after the disconnection from the ventilator. Opposite to previous Clini’s study results [29], none of our patients required bronchoscopic procedures for secretion clearance and they all have been successfully weaned from MV. Discrepancy in results could be related to the time interval when the cycle was delivered. Specifically, Clini et al. applied the cycle every 12 h [29], whereas we halved the interval to 6 h. This may suggest benefits of HFPV when applied more frequently, however designed studies are required to prove this hypothesis. Our study also has limitations which must be addressed.

Firstly, the sample size population. Given the physiological design of this study, a formal sample size calculation has not been performed. Despite the total number of enrolled patients (15) being relatively small and the population is quite heterogeneous, several studies enrolled similar or even lower numbers of patients [20, 21, 36, 42]. Hence, we considered 15 patients as sufficient to address our hypothesis based on our previous trial [7]. In addition, we assumed all our data as non-parametric.

Secondly, the definition of hypersecretive patient could be arguable. We adopted the same definition mentioned in previous studies [7, 43, 44] as other criteria for definition of hypersecretive status have been considered in literature but they seem to be more operator dependent, hence open to criticism [45].

Thirdly, the lack of a standardised bronchoaspiration protocol. In other words, the clinical evaluation of the single operator (nurse) in deciding how often suctions were needed within 8 h could have generated a bias in the patient’s selection.

Last, we expressed variation in EIT volumes (TIV and ∆EELI) in mL, instead of arbitrary units. It should be mentioned that EIT device we used, measures an eclipse with a central diameter of 5–10 cm [20]. Therefore, the assessment is limited to a 5–10 cm slice of the lung, rather than the entire lung. Noteworthy, this method has been previously extensively used in the literature [7, 16, 17].

The use of HFPV superimposed to MV is promising, however additional evidence on less selected populations are needed.

## Supplementary Information

Below is the link to the electronic supplementary material.

Supplementary file1 (DOCX 32 kb)

## References

[1] Sackner MA, Hirsch J, Epstein S (1975). Effect of cuffed endotracheal tubes on tracheal mucous velocity. Chest.

[2] Konrad F, Schreiber T, Brecht-Kraus D, Georgieff M (1994). Mucociliary transport in ICU patients. Chest.

[3] Konrad F, Schreiber T, Grunert A, Clausen M, Ahnefeld FW (1992). Measurement of mucociliary transport velocity in ventilated patients. Short-term effect of general anesthesia on mucociliary transport. Chest.

[4] Williams R, Rankin N, Smith T, Galler D, Seakins P (1996). Relationship between the humidity and temperature of inspired gas and the function of the airway mucosa. Crit Care Med.

[5] Pneumatikos IA, Dragoumanis CK, Bouros DE (2009). Ventilator-associated pneumonia or endotracheal tube-associated pneumonia? An approach to the pathogenesis and preventive strategies emphasizing the importance of endotracheal tube. Anesthesiology.

[6] Jelic S, Cunningham JA, Factor P (2008). Clinical review: airway hygiene in the intensive care unit. Crit Care.

[7] Longhini F, Bruni A, Garofalo E, Ronco C, Gusmano A, Cammarota G, Pasin L, Frigerio P, Chiumello D, Navalesi P (2020). Chest physiotherapy improves lung aeration in hypersecretive critically ill patients: a pilot randomized physiological study. Crit Care.

[8] Hodgson C, Carroll S, Denehy L (1999). A survey of manual hyperinflation in Australian hospitals. Aust J Physiother.

[9] Mackenzie CF, Shin B (1985). Cardiorespiratory function before and after chest physiotherapy in mechanically ventilated patients with post-traumatic respiratory failure. Crit Care Med.

[10] MacLean D, Drummond G, Macpherson C, McLaren G, Prescott R (1989). Maximum expiratory airflow during chest physiotherapy on ventilated patients before and after the application of an abdominal binder. Intensive Care Med.

[11] Ntoumenopoulos G, Presneill JJ, McElholum M, Cade JF (2002). Chest physiotherapy for the prevention of ventilator-associated pneumonia. Intensive Care Med.

[12] Trawoger R, Kolobow T, Cereda M, Giacomini M, Usuki J, Horiba K, Ferrans VJ (1997). Clearance of mucus from endotracheal tubes during intratracheal pulmonary ventilation. Anesthesiology.

[13] Natale JE, Pfeifle J, Homnick DN (1994). Comparison of intrapulmonary percussive ventilation and chest physiotherapy. A pilot study in patients with cystic fibrosis. Chest.

[14] Toussaint M, De Win H, Steens M, Soudon P (2003). Effect of intrapulmonary percussive ventilation on mucus clearance in duchenne muscular dystrophy patients: a preliminary report. Respir Care.

[15] AARC Clinical Practice Guidelines (2010). Endotracheal suctioning of mechanically ventilated patients with artificial airways 2010. Respir Care.

[16] Longhini F, Pelaia C, Garofalo E, Bruni A, Placida R, Iaquinta C, Arrighi E, Perri G, Procopio G, Cancelliere A, Rovida S, Marrazzo G, Pelaia G, Navalesi P (2022). High-flow nasal cannula oxygen therapy for outpatients undergoing flexible bronchoscopy: a randomised controlled trial. Thorax.

[17] Longhini F, Maugeri J, Andreoni C, Ronco C, Bruni A, Garofalo E, Pelaia C, Cavicchi C, Pintaudi S, Navalesi P (2019). Electrical impedance tomography during spontaneous breathing trials and after extubation in critically ill patients at high risk for extubation failure: a multicenter observational study. Ann Intensive Care.

[18] Yang L, Qu S, Zhang Y, Zhang G, Wang H, Yang B, Xu C, Dai M, Cao X (2022). Removing clinical motion artifacts during ventilation monitoring with electrical impedance tomography: introduction of methodology and validation with simulation and patient data. Front Med (Lausanne).

[19] Frerichs I, Pulletz S, Elke G, Gawelczyk B, Frerichs A, Weiler N (2011). Patient examinations using electrical impedance tomography–sources of interference in the intensive care unit. Physiol Meas.

[20] Bikker IG, Preis C, Egal M, Bakker J, Gommers D (2011). Electrical impedance tomography measured at two thoracic levels can visualize the ventilation distribution changes at the bedside during a decremental positive end-expiratory lung pressure trial. Crit Care.

[21] Mauri T, Eronia N, Abbruzzese C, Marcolin R, Coppadoro A, Spadaro S, Patroniti N, Bellani G, Pesenti A (2015). Effects of sigh on regional lung strain and ventilation heterogeneity in acute respiratory failure patients undergoing assisted mechanical ventilation. Crit Care Med.

[22] Zhao Z, Wang W, Zhang Z, Xu M, Frerichs I, Wu J, Moeller K (2018). Influence of tidal volume and positive end-expiratory pressure on ventilation distribution and oxygenation during one-lung ventilation. Physiol Meas.

[23] Marusich LR, Bakdash JZ (2021). rmcorrShiny: a web and standalone application for repeated measures correlation. F1000Res.

[24] Brennan M, McDonnell MJ, Duignan N, Gargoum F, Rutherford RM (2022). The use of cough peak flow in the assessment of respiratory function in clinical practice—A narrative literature review. Respir Med.

[25] De Pascale G, Ranzani OT, Nseir S, Chastre J, Welte T, Antonelli M, Navalesi P, Garofalo E, Bruni A, Coelho LM, Skoczynski S, Longhini F, Taccone FS, Grimaldi D, Salzer HJF, Lange C, Froes F, Artigas A, Diaz E, Valles J, Rodriguez A, Panigada M, Comellini V, Fasano L, Soave PM, Spinazzola G, Luyt CE, Alvarez-Lerma F, Marin J, Masclans JR, Chiumello D, Pezzi A, Schultz M, Mohamed H, Van Der Eerden M, Hoek RAS, Gommers D, Pasquale MD, Civljak R, Kutlesa M, Bassetti M, Dimopoulos G, Nava S, Rios F, Zampieri FG, Povoa P, Bos LD, Aliberti S, Torres A, Martin-Loeches I (2017). Intensive care unit patients with lower respiratory tract nosocomial infections: the ENIRRIs project. ERJ Open Res.

[26] Frigerio P, Longhini F, Sommariva M, Stagni EG, Curto F, Redaelli T, Ciboldi M, Simonds AK, Navalesi P (2015). Bench comparative assessment of mechanically assisted cough devices. Respir Care.

[27] Morrison L, Innes S (2017). Oscillating devices for airway clearance in people with cystic fibrosis. Cochrane Database Syst Rev.

[28] Salim A, Martin M (2005). High-frequency percussive ventilation. Crit Care Med.

[29] Clini EM, Antoni FD, Vitacca M, Crisafulli E, Paneroni M, Chezzi-Silva S, Moretti M, Trianni L, Fabbri LM (2006). Intrapulmonary percussive ventilation in tracheostomized patients: a randomized controlled trial. Intensive Care Med.

[30] Tsuruta R, Kasaoka S, Okabayashi K, Maekawa T (2006). Efficacy and safety of intrapulmonary percussive ventilation superimposed on conventional ventilation in obese patients with compression atelectasis. J Crit Care.

[31] Godet T, Jabaudon M, Blondonnet R, Tremblay A, Audard J, Rieu B, Pereira B, Garcier JM, Futier E, Constantin JM (2018). High frequency percussive ventilation increases alveolar recruitment in early acute respiratory distress syndrome: an experimental, physiological and CT scan study. Crit Care.

[32] Zhao Z, Moller K, Steinmann D, Frerichs I, Guttmann J (2009). Evaluation of an electrical impedance tomography-based global inhomogeneity index for pulmonary ventilation distribution. Intensive Care Med.

[33] Schullcke B, Krueger-Ziolek S, Gong B, Jorres RA, Mueller-Lisse U, Moeller K (2018). Ventilation inhomogeneity in obstructive lung diseases measured by electrical impedance tomography: a simulation study. J Clin Monit Comput.

[34] Zhang W, Liu F, Zhao Z, Shao C, Xu X, Ma J, Han R (2022). Driving pressure-guided ventilation improves homogeneity in lung gas distribution for gynecological laparoscopy: a randomized controlled trial. Sci Rep.

[35] Liu X, Meng J, Liu D, Huang Y, Sang L, Xu Y, Xu Z, He W, Chen S, Zhang R, Li Y (2022). Electrical impedance tomography for titration of positive end-expiratory pressure in acute respiratory distress syndrome patients with chronic obstructive pulmonary disease. Crit Care.

[36] Zhao Z, Steinmann D, Frerichs I, Guttmann J, Moller K (2010). PEEP titration guided by ventilation homogeneity: a feasibility study using electrical impedance tomography. Crit Care.

[37] Heines SJH, de Jongh SAM, Strauch U, van der Horst ICC, van de Poll MCG, Bergmans D (2022). The global inhomogeneity index assessed by electrical impedance tomography overestimates PEEP requirement in patients with ARDS: an observational study. BMC Anesthesiol.

[38] Chi Y, Zhao Z, Frerichs I, Long Y, He H (2022). Prevalence and prognosis of respiratory pendelluft phenomenon in mechanically ventilated ICU patients with acute respiratory failure: a retrospective cohort study. Ann Intensive Care.

[39] Sang L, Zhao Z, Yun PJ, Frerichs I, Moller K, Fu F, Liu X, Zhong N, Li Y (2020). Qualitative and quantitative assessment of pendelluft: a simple method based on electrical impedance tomography. Ann Transl Med.

[40] Coppadoro A, Grassi A, Giovannoni C, Rabboni F, Eronia N, Bronco A, Foti G, Fumagalli R, Bellani G (2020). Occurrence of pendelluft under pressure support ventilation in patients who failed a spontaneous breathing trial: an observational study. Ann Intensive Care.

[41] Boles JM, Bion J, Connors A, Herridge M, Marsh B, Melot C, Pearl R, Silverman H, Stanchina M, Vieillard-Baron A, Welte T (2007). Weaning from mechanical ventilation. Eur Respir J.

[42] Lowhagen K, Lundin S, Stenqvist O (2010). Regional intratidal gas distribution in acute lung injury and acute respiratory distress syndrome–assessed by electric impedance tomography. Minerva Anestesiol.

[43] Vaschetto R, Longhini F, Persona P, Ori C, Stefani G, Liu S, Yi Y, Lu W, Yu T, Luo X, Tang R, Li M, Li J, Cammarota G, Bruni A, Garofalo E, Jin Z, Yan J, Zheng R, Yin J, Guido S, Della Corte F, Fontana T, Gregoretti C, Cortegiani A, Giarratano A, Montagnini C, Cavuto S, Qiu H, Navalesi P (2019). Early extubation followed by immediate noninvasive ventilation vs. standard extubation in hypoxemic patients: a randomized clinical trial. Intensive Care Med.

[44] Navalesi P, Frigerio P, Moretti MP, Sommariva M, Vesconi S, Baiardi P, Levati A (2008). Rate of reintubation in mechanically ventilated neurosurgical and neurologic patients: evaluation of a systematic approach to weaning and extubation. Crit Care Med.

[45] Khamiees M, Raju P, DeGirolamo A, Amoateng-Adjepong Y, Manthous CA (2001). Predictors of extubation outcome in patients who have successfully completed a spontaneous breathing trial. Chest.

