# Supplementary material for: Benefits of secretion clearance with high frequency percussive ventilation in tracheostomized critically ill patients: a pilot study
Source: J Clin Monit Comput. 2023 Jan 6;37(3):911–8. doi: 10.1007/s10877-022-00970-7 (PMC10175357; doi:10.1007/s10877-022-00970-7)
Supplement: Supplementary file 1 — Supplementary file1 (DOCX 32 kb) [file 10877_2022_970_MOESM1_ESM.docx]

**ONLINE SUPPLEMENTAL MATERIAL**

**Benefits of secretion clearance with High Frequency Percussive Ventilation in tracheostomized critically ill patients: A pilot study.**

Eugenio Garofalo, MD^1#^; Serena Rovida, MD^2#^; Gianmaria Cammarota, MD PhD^3^; Eugenio Biamonte, MD^1^; Letizia Troisi, MD^1^; Leonardo Cosenza, MD^1^; Corrado Pelaia, MD^4^; Paolo Navalesi, MD, FERS^5^; Federico Longhini, MD^1^; Andrea Bruni, MD^1^.

^1^Anaesthesia and Intensive Care, Department of Medical and Surgical Sciences, “Magna Graecia” University, Catanzaro, Italy; ^2^Department of Intensive Care Medicine, St George's University Hospital, London, United Kingdom; ^3^Department of Anaesthesia and Intensive Care Medicine, University of Perugia, Perugia, Italy; ^4^Pulmonary Medicine Unit, Department of Health Sciences, “Magna Graecia” University, Catanzaro, Italy; ^5^Anaesthesia and Intensive Care, Padua Hospital, Department of Medicine - DIMED, University of Padua, Italy.

^#^These authors contributed equally to the work

​​Corresponding author:

Prof. Federico Longhini, MD

Intensive Care Unit, “Mater Domini” University Hospital,

Department of Medical and Surgical Sciences,

Magna Graecia University, Viale Europa, 88100, Catanzaro, Italy

E-mail: longhini.federico@gmail.com

Tel: +393475395967

**Data acquisition and analysis**

Data were recorded by the EIT system at a sample of 20 Hz, coupled to data imported from the ventilator (*i.e.;* airway pressure, flow, tidal volume); afterwards, data were downloaded on a USB memory stick to be off line analyzed on a personal computer [1-3]. Analysis was performed with dedicated software (EITdiag, Draeger Medical GmbH, Lübeck, Germany). The last three minutes of each record were analyzed. A "DATlowpass" filter was set at values higher than the patient's heart rate to purify the signal from the cardiac oscillation. EIT scans are represented by 32 × 32 color-coded matrix images showing impedance relative to the lowest recorded value (relative ΔZ) [4]. We measured the tidal impedance variation (TIV) as the difference between the relative ΔZ at the end of inspiration and expiration [5,4]. Tidal impedance changes were calibrated against known lung volume imported by the ventilator. Changes in end-expiratory lung impedance (ΔEELI, mL) from baseline (T0) were also computed at T1, T2 and T3 [1,6-7]. We also defined two contiguous regions of interest (ROIs) of the same size (ventral and dorsal) and computed TIV and ΔEELI for both [3,8-9]. We also computed the inhomogeneity index (GI) to assess the gas distribution within the lung [10].

**Table E1 – Vital Parameters and Arterial Blood Gases**

| **Data** | **T0** | **T1** | **T2** | **T3** | **P value** |
| --- | --- | --- | --- | --- | --- |
| ***Vital Parameters*** |  |  |  |  |  |
| Heart rate (beat/min) | 81 [76; 90] | 83 [78; 90] | 84 [80; 89] | 82 [80; 88] | 0.326 |
| Mean Arterial Pressure (mmHg) | 80 [75; 88] | 85 [81; 89] | 82 [79; 88] | 85 [83; 91] | 0.085 |
| Respiratory Rate (breath/min) | 19 [16; 22] | 18 [16; 21] | 18 [16; 20] | 18 [16; 21] | 0.078 |
| ***Arterial Blood Gases*** |  |  |  |  |  |
| pH | 7.44 [7.40; 7.46] | 7.45 [7.39; 7.47] | 7.46 [7.40; 7.47] | 7.46 [7.40; 7.47] | 0.226 |
| PaCO_2_ (mmHg) | 45 [39; 50] | 45 [39; 47] | 44 [37; 45] | 44 [36; 47] | 0.114 |
| PaO_2_/FiO_2_ (mmHg) | 174 [171; 185] | 211 [198; 225]^a^ | 230 [213; 251]^b^ | 225 [203; 251]^c^ | <0.001 |

T0, baseline assessment; T1, assessment soon after the end of the treatment; T2, assessment 1 hour after the end of the treatment; T3, assessment 3 hours after the end of the treatment; PaCO_2_, arterial partial pressure of carbon dioxide; PaO_2_/FiO_2_, ratio between arterial partial pressure and inspired fraction of oxygen. All data are expressed as median [25^th^-75^th^ percentile].

^a^ p<0.001 T1 vs T0; ^b^ p<0.001 T2 vs. T0; ^c^ p<0.001 T3 vs. T0.

**SUPPLEMENTAL REFERENCES**

1. Longhini F, Bruni A, Garofalo E, Ronco C, Gusmano A, Cammarota G, Pasin L, Frigerio P, Chiumello D, Navalesi P (2020) Chest physiotherapy improves lung aeration in hypersecretive critically ill patients: a pilot randomized physiological study. Crit Care 24 (1):479. doi:10.1186/s13054-020-03198-6

10.1186/s13054-020-03198-6 [pii]

2. Longhini F, Pelaia C, Garofalo E, Bruni A, Placida R, Iaquinta C, Arrighi E, Perri G, Procopio G, Cancelliere A, Rovida S, Marrazzo G, Pelaia G, Navalesi P (2022) High-flow nasal cannula oxygen therapy for outpatients undergoing flexible bronchoscopy: a randomised controlled trial. Thorax 77 (1):58-64. doi:thoraxjnl-2021-217116 [pii]

10.1136/thoraxjnl-2021-217116

3. Longhini F, Maugeri J, Andreoni C, Ronco C, Bruni A, Garofalo E, Pelaia C, Cavicchi C, Pintaudi S, Navalesi P (2019) Electrical impedance tomography during spontaneous breathing trials and after extubation in critically ill patients at high risk for extubation failure: a multicenter observational study. Ann Intensive Care 9 (1):88. doi:10.1186/s13613-019-0565-0 [pii]

565 [pii]

10.1186/s13613-019-0565-0

4. Costa EL, Lima RG, Amato MB (2009) Electrical impedance tomography. Curr Opin Crit Care 15 (1):18-24. doi:10.1097/mcc.0b013e3283220e8c

5. Moerer O, Hahn G, Quintel M (2011) Lung impedance measurements to monitor alveolar ventilation. Curr Opin Crit Care 17 (3):260-267. doi:10.1097/MCC.0b013e3283463c9c

6. Lowhagen K, Lindgren S, Odenstedt H, Stenqvist O, Lundin S (2011) A new non-radiological method to assess potential lung recruitability: a pilot study in ALI patients. Acta Anaesthesiol Scand 55 (2):165-174. doi:10.1111/j.1399-6576.2010.02331.x

7. Frerichs I, Schmitz G, Pulletz S, Schadler D, Zick G, Scholz J, Weiler N (2007) Reproducibility of regional lung ventilation distribution determined by electrical impedance tomography during mechanical ventilation. Physiol Meas 28 (7):S261-267. doi:S0967-3334(07)36585-4 [pii]

10.1088/0967-3334/28/7/S19

8. Mauri T, Eronia N, Abbruzzese C, Marcolin R, Coppadoro A, Spadaro S, Patroniti N, Bellani G, Pesenti A (2015) Effects of Sigh on Regional Lung Strain and Ventilation Heterogeneity in Acute Respiratory Failure Patients Undergoing Assisted Mechanical Ventilation. Crit Care Med 43 (9):1823-1831. doi:10.1097/CCM.0000000000001083

9. Dargaville PA, Rimensberger PC, Frerichs I (2010) Regional tidal ventilation and compliance during a stepwise vital capacity manoeuvre. Intensive Care Med 36 (11):1953-1961. doi:10.1007/s00134-010-1995-1

10. Zhao Z, Wang W, Zhang Z, Xu M, Frerichs I, Wu J, Moeller K (2018) Influence of tidal volume and positive end-expiratory pressure on ventilation distribution and oxygenation during one-lung ventilation. Physiol Meas 39 (3):034003. doi:10.1088/1361-6579/aaaeb2
